# A 2D and 3D cell culture protocol to study O-GlcNAc in sphingosine-1-phosphate mediated fibroblast contraction

**DOI:** 10.1016/j.xpro.2021.101113

**Published:** 2022-01-20

**Authors:** Murielle M. Morales, Nichole J. Pedowitz, Matthew R. Pratt

**Affiliations:** 1 Departments of Chemistry, University of Southern California, Los Angeles, CA 90089, USA; 2 Biological Sciences, University of Southern California, Los Angeles, CA 90089, USA

**Keywords:** Biophysics, Cell Biology, Cell culture, Cell-based Assays, Microscopy, Molecular/Chemical Probes

## Abstract

O-linked β-N-acetylglucosamine (O-GlcNAc) is an abundant posttranslational modification involved in a wide range of signaling pathways, but its specific role in regulating biological processes remains unclear. This protocol describes approaches to understand O-GlcNAc’s role in fibroblast contraction. Specifically, cellular O-GlcNAc levels are controlled through treatment of fibroblasts with inhibitors in both 2D and 3D cultures. We then describe 2D contraction assay and 3D collagen gel contraction assay to analyze the effect of the modification on sphingosine-1-phosphate signaling for contraction.

For complete details on the use and execution of this protocol, please refer to Pedowitz et al. (2021).

## Highlights

• Protocol describes approaches to test fibroblast contraction in 2D and 3D cell cultures
• Fibroblast contraction is induced by the signaling lipid sphingosine-1-phosphate (S1P)
• Extent of S1P-induced contraction is controlled by O-GlcNAc modification
• Protocol can be used to test a variety of established and potential contraction mediators

## Graphical abstract

**Figure:**
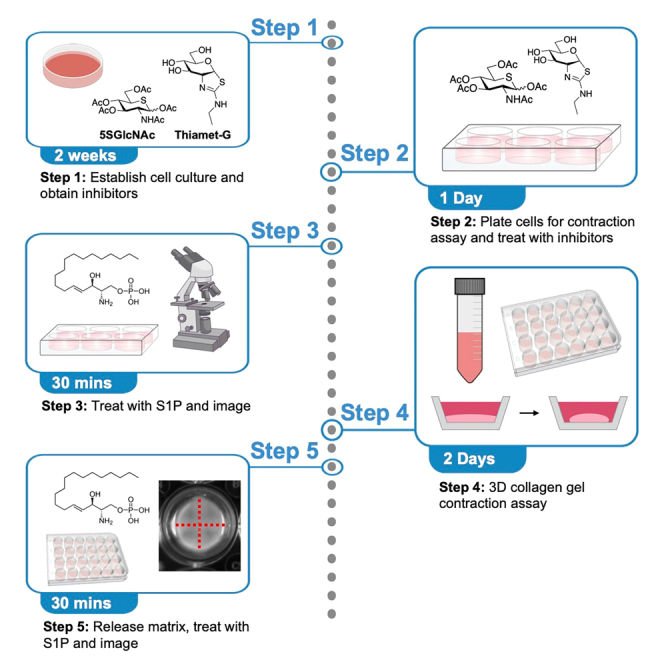

## Before you begin

The protocol below describes the method to study the effect of O-GlcNAc on sphingosine-1-phosphate (S1P) mediated 2D cell contraction in mouse embryonic fibroblast NIH3T3 cells and 3D cell contraction in primary human dermal fibroblast BJ-5ta cells. We have also used this protocol to look at signaling by lysophosphatidic acid (LPA) (Morales et al., 2021).

Small molecule inhibitors Thiamet-G (Yuzwa et al., 2008) and Ac_4_5SGlcNAc (Gloster et al., 2011) can be synthesized according to literature procedures. Thiamet-G (Sigma Aldrich, SML0244) is also commercially available. An alternative OGT inhibitor (OSMI-4) (Martin et al., 2018) is also commercially available (Selleck Chem, S8910).

### Synthesis of small molecule inhibitors

**Timing: 2 weeks**

1. Synthesize known compounds Thiamet-G (Yuzwa et al., 2008) and Ac_4_5SGlcNAc (Gloster et al., 2011) according to literature procedures. Dissolve both inhibitors as 1,000 X stocks in DMSO, with Thiamet-G at 10 mM and 5SGlcNAc at 200 mM.

## Thawing and culturing NIH3T3 and BJ-5ta cells

**Timing: 1 week**

2. Thawing NIH3T3 or BJ-5ta cells
  a. Obtain a frozen NIH3T3 or BJ-5ta cell stock.
  b. Briefly thaw frozen cells at 37°C until only a small amount of ice remains.
  c. In a biological safety hood, transfer the cells into a 15 mL tube containing 10 mL of its appropriate pre-warmed culture medium.
  d. Centrifuge at 500 *g* for 5 min.
  e. Discard the supernatant without disturbing the cell pellet.
  f. Gently resuspend the cell pellet in the appropriate culture medium and transfer into a tissue culture plate.
3. Maintaining NIH3T3 or BJ-5ta cells
  a. Subculture cells every 2–3 days or until 80% confluent and replenish media every 3 days.

**CRITICAL:** Subculture NIH3T3 cells at least 3 times before continuing with contraction experiment and use before a maximum of 6 passages. Subculture BJ-5ta cells at least twice before experiment; however, do not use BJ-5ta cells that have been passaged more than 8 times.

## Key resources table

**Table:** 

REAGENT or RESOURCE	SOURCE	IDENTIFIER
**Chemicals, peptides, and recombinant proteins**		

DMEM, High Glucose, with L-Glutamine and with Sodium Pyruvate	Genesee Scientific	25-500
Trypsin-EDTA, 0.25% 1×, phenol red	Genesee Scientific	25-510
Iron-Supplemented Calf Serum	Atlanta Biologicals	S11950
Fetal bovine serum	R&D Systems	S11150
Hygromycin B	VWR	97064-810
Sphingosine-1-phosphate	Cayman Chemical	62570
Dimethyl sulfoxide	VWR	MK494802
Thiamet-G	Sigma-Aldrich	SML0244

**Antibodies**		

Anti-RL2 (1:1000)	Thermo Fisher Scientific	MA1-072

**Experimental models: Cell lines**		

NIH/3T3 Cell Line	Cedarlane	EP-CL-0171
BJ-5ta	ATCC	CRL-4001

**Critical commercial assays**		

Cell Contraction Assays, Two-Step Attached Model	Cell Biolabs	CBA-201

**Software and algorithms**		

ImageJ	National Institutes of Health	1.53k
Photoshop	Adobe	23.0.2

**Other**		

6-Well Cell Culture Plates	Genesee Scientific	25-205
24-Well Cell Culture Plates	Genesee Scientific	25-107

## Materials and equipment

**Table:** NIH3T3 culture medium

Reagent	Final concentration	Amount
DMEM	n/a	500 mL
Calf Serum	10%	50 mL
**Total**	**n/a**	**550 mL**

Store medium at 4°C and use within 1 month.

**Table:** BJ-5ta culture medium

Reagent	Final concentration	Amount
DMEM	n/a	500 mL
Fetal Bovine Serum	10%	50 mL
Hygromycin B	0.01 mg mL^-1^	5.5 mg
**Total**	**n/a**	**550 mL**

Store medium at 4°C and use within 1 month.

**CRITICAL:** ATCC recommends that BJ-5ta cells are grown in a 4:1 mixture of Dulbecco's medium and Medium 199 with supplements, however we have seen that BJ-5ta cells grow equally well in unmixed DMEM.

**Table:** Collagen solution (Cell Biolabs kit)

Reagent	Final concentration	Amount
Collagen Solution	n/a	10 mL
5× Medium	n/a	2.58 mL
Neutralization Solution	n/a	0.356 mL
**Total**	**n/a**	**12.936 mL**

Keep on ice and prepare immediately before use.

## Step-by-step method details

### 2D contraction assay using NIH3T3 cells

**Timing: 2 days**

**Figure 1:**
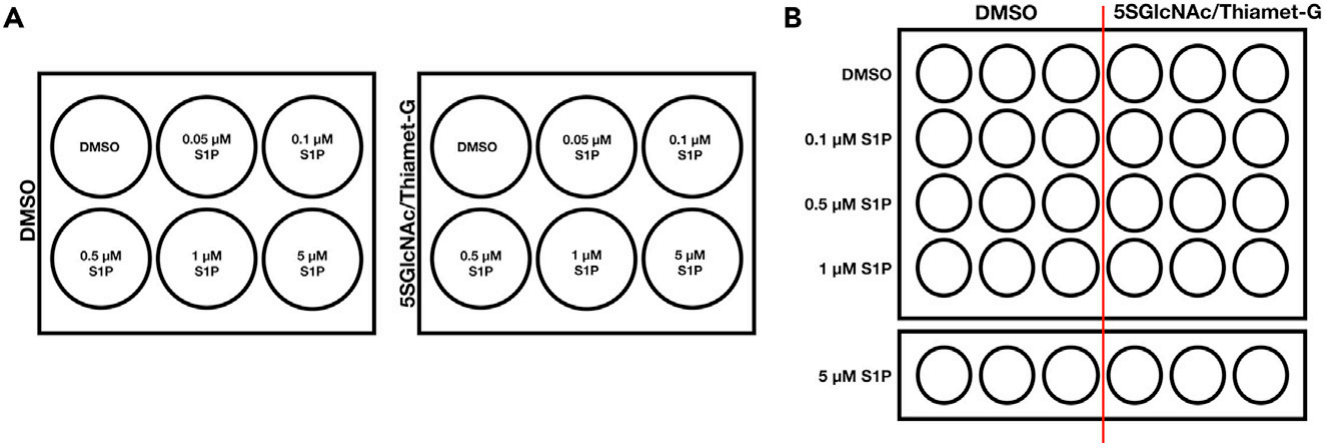
Layout of the 6-well plates and the 24-well plates for the 2D and 3D contraction assays (A) A schematic of the treatment of inhibitors and different concentrations of sphingosine-1-phosphate on NIH3T3 cells in the 2D contraction assay. (B) A schematic of the treatment of inhibitors and different concentrations of sphingosine-1-phosphate on BJ-5ta cells in the 3D collagen gel contraction assay.

This step contains information on plating NIH3T3 cells, and treatment of the cells with either inhibitor and sphingosine-1-phosphate to initiate contraction. Figure 1A shows a diagram of the layout of the different cell treatments.

1. Seed NIH3T3 cells at 1 × 10^5^ cells per well in 12 wells of two 6-well cell culture dishes 8 h prior to treatment with either small molecule inhibitors.
2. After 8 h, treat a 6-well dish of NIH3T3 cells with either DMSO vehicle, 5SGlcNAc (200 μM), or Thiamet-G (10 μM).
  a. Allow cells to incubate for 16 h with 5SGlcNAc/DMSO or for 20 h with Thiamet-G/DMSO.
3. After the appropriate incubation, treat 1 well with DMSO as control and treat 5 wells of each plate with a S1P dose curve (0.05 μM, 0.1 μM, 0.5 μM, 1 μM, 5 μM) by pipetting it into the media (Figure 1A). Incubate the plate for 30 min.
4. After 30 min, take 5 representative images throughout each well by brightfield microscopy at 20× magnification.
5. Analyze the images by Adobe photoshop to find the change in cell area (see quantification protocol below).

### 3D contraction assay using BJ-5ta cells

**Timing: 3 days**

This step contains information on using a collagen gel contraction assay to evaluate the effect of inhibitors and sphingosine-1-phosphate on cell contraction in a tissue like environment. Figure 1B shows a diagram of the layout of the different cell treatments. This protocol was adapted from a procedure that can be found on the manufacturer’s website: https://www.cellbiolabs.com/cell-contraction-two-step-attached-model.

6. Suspend at least 7 × 10^6^ BJ-5ta cells in fresh media at 2 × 10^6^ cells per mL.
7. In a pre-chilled falcon tube, prepare the collagen solution on ice according to the ‘Collagen solution’ table above.
  a. First add the collagen solution and 5× medium and mix well by gently pipetting up and down.
  b. Add the neutralization solution and mix immediately.

**CRITICAL:** Keep everything on ice to prevent premature polymerization of the solution. Make the solution right before plating out for the experiment.

8. Make a 4:1 mixture of collagen stock solution to cells in a pre-chilled 15 mL falcon tube and mix well.
  a. To 12.936 mL of collagen solution, add 3.23 mL of BJ-5ta cells in media.
  b. Pipette up and down carefully to avoid bubbles. Do not vortex.
9. Plate 500 μL of the cell collagen solution into 30 wells in two 24-well dishes.
10. Incubate the cell-collagen matrix at 37°C with 5% CO_2_ for 1 h to allow collagen polymerization to occur.

***Note:*** It is difficult to determine whether polymerization is successful. Figure 7 shows a representative image of successful vs unsuccessful polymerization. Commonly, we discovered polymerization was unsuccessful when there was no contraction upon the addition of S1P.

11. After 1 h, add 1 mL of prewarmed media to each well.

**Figure 2:**
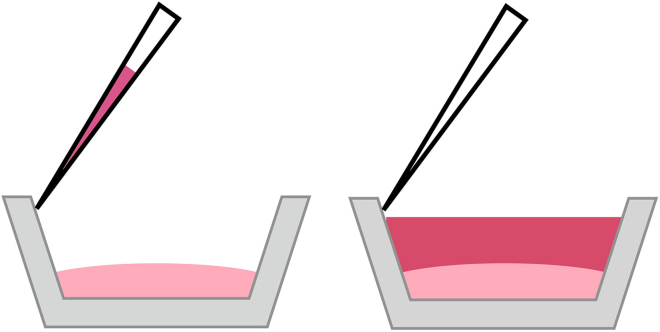
How to carefully add media to the wells A diagram showing how to correctly pipette 1 mL of media against the wall of the well onto the cell collagen matrix gently so as not to disturb the forming matrix (step 11).

**CRITICAL:** Add the media very gently to prevent the matrix from detaching from the plate. Using a pipet, press the pipet tip against the wall of the well and depress the plunger slowly (Figure 2).

12. Incubate the matrix at 37°C with 5% CO_2_ for 30 h.
13. After 30 h, supplement the wells with DMSO vehicle or with either 5SGlcNAc (200 μM) or Thiamet-G (10 μM).
  a. Treat 15 wells with DMSO vehicle and 15 wells with 5SGlcNAc/Thiamet-G.
14. Incubate for an additional 16 h.
15. After incubation, carefully aspirate the liquid media out of the well and replace the media with serum-free media containing S1P (0.1–5 μM) or no S1P as a control.
  a. Treat 6 wells with each concentration of S1P: 3 wells that were pre-treated with DMSO and 3 wells that were pre-treated with 5SGlcNAc.
  b. Treat one row of 6 wells with DMSO as control.
16. Initiate contraction by gently releasing the sides and the bottom of the matrix from the plate using a 10 μL pipet tip.
17. Image the wells using a ChemiDoc XRS+ molecular imager (Bio-Rad) immediately after release (t = 0). Alternatively, images can be taken with any type of imager or the matrix can be measured with a ruler.
18. Incubate the matrix at 37°C for 30 min and then image the wells again (t = 30).
19. Quantify the change in diameter of each matrix using ImageJ (see quantification protocol below).

## Expected outcomes

**Figure 3:**
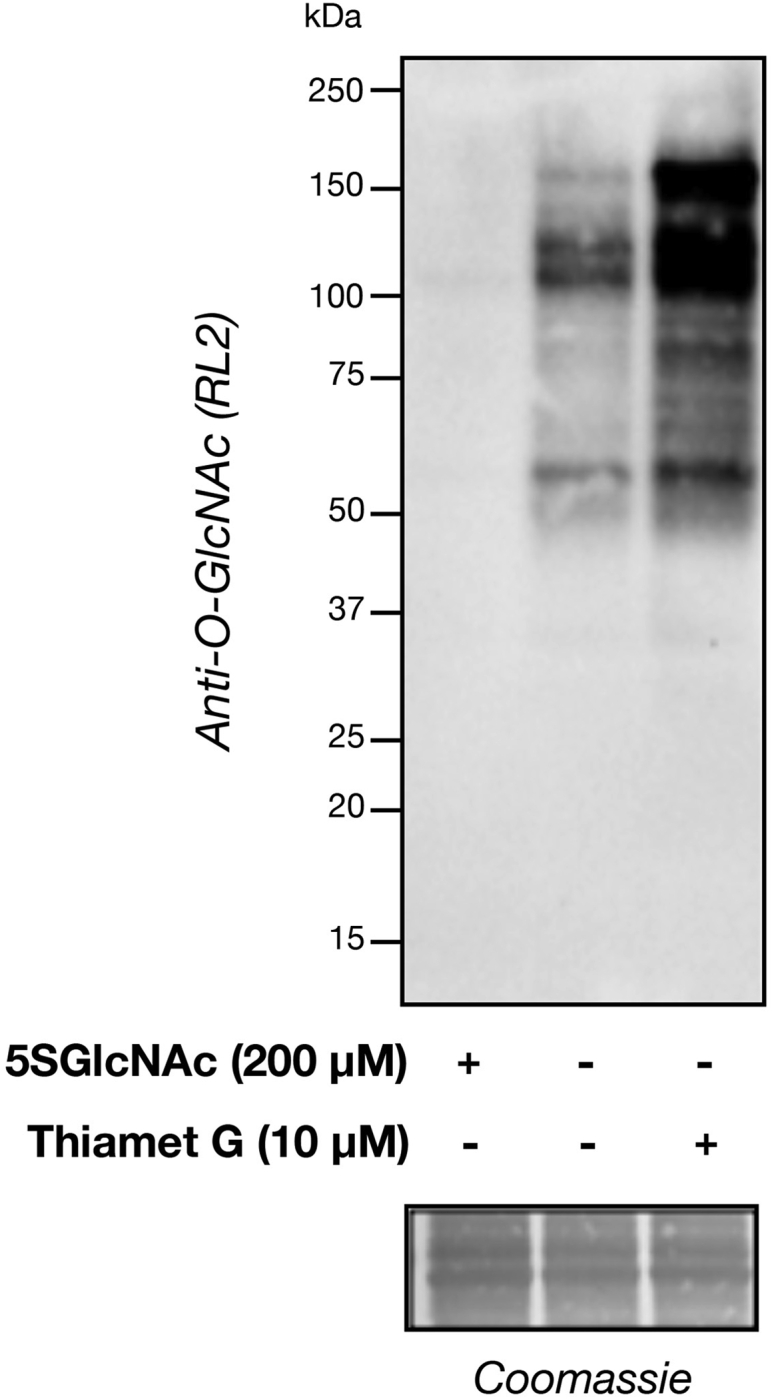
O-GlcNAc levels can be modulated through treatment with inhibitors NIH3T3 cells were treated with either 5SGlcNAc (200 μM, 16 h), DMSO, or Thiamet G (10 μM, 20 h) before analysis of O-GlcNAc levels by western blotting. Proteins were separated by SDS-PAGE and transferred to a PDVF membrane. The blot was then blocked in OneBlock Western-CL blocking buffer (Genesee) for 1 h at room temperature (RT; 20°C–25°C) then incubated with primary antibody RL2 (1:1,000 dilution) in fresh blocking buffer at 4°C overnight (14–18 h). Following overnight incubation, blots were washed in TBST (Cell Signaling, 3 × 10 min) and incubated with HRP-conjugated secondary antibody (1:10,000 dilution) in fresh blocking buffer for 1 h at RT then washed again with TBST. Blots were developed using ECL reagents (Bio-Rad) and the ChemiDoc XRS+ molecular imager.

**Figure 4:**
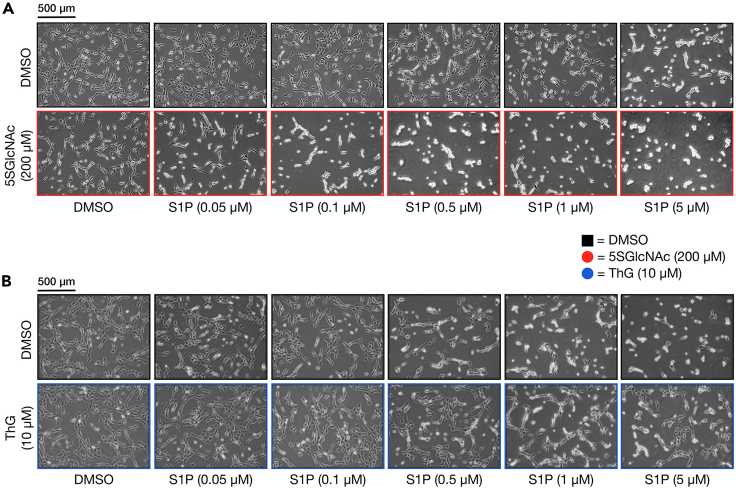
O-GlcNAc controls the sensitivity of cells to S1P-mediated contraction (A and B) NIH3T3 cells were pre-treated with either DMSO or 5SGlcNAc (A) or with Thiamet-G (B) before addition of the indicated concentrations of S1P. The contraction phenotype was then visualized using bright-field microscopy.

**Figure 5:**
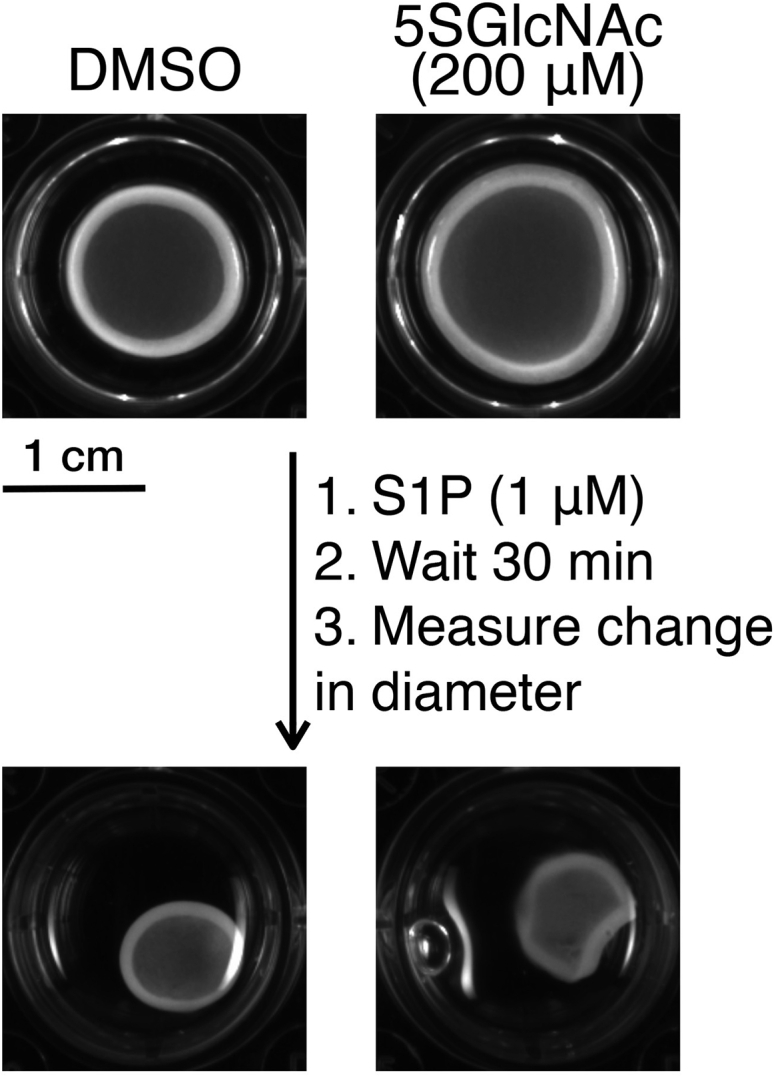
O-GlcNAc controls the sensitivity of human dermal fibroblasts to S1P-mediated contraction in 3D collagen matrices BJ-5ta cells were plated with a collagen solution and allowed to polymerize before being pre-treated with either DMSO or 5SGlcNAc. The matrices were then released from the plate and contraction was initiated by the addition of S1P. Images were taken after release of the matrix then after 30 min of incubation with S1P.

The modulation of O-GlcNAc levels through treatment with inhibitors can be confirmed by western blotting (Figure 3). For the 2D contraction assay, NIH3T3s with normal levels of O-GlcNAc undergo contraction at higher concentrations of S1P. However, NIH3T3s treated with OGT inhibitor 5SGlcNAc with lower levels of O-GlcNAc displayed increased sensitivity to lower concentrations of S1P (Figure 4A). Conversely, NIH3T3s that were treated with OGA inhibitor Thiamet-G and had higher O-GlcNAc levels were more resistant to S1P-mediated contraction compared to control DMSO treated cells (Figure 4B). Similarly in the 3D contraction assay with BJ-5ta cells, the matrices that were pretreated with 5SGlcNAc were significantly more sensitive to S1P-mediated contraction than the control (Figure 5).

## Quantification and statistical analysis

### 2D contraction assay using NIH3T3 cells

**Figure 6:**
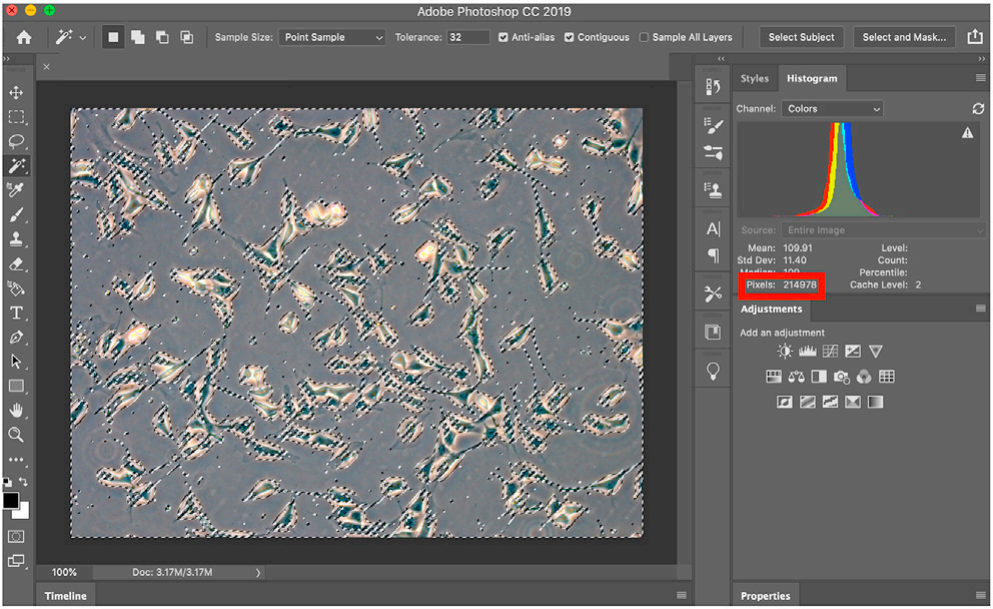
Quantification of cell area using Adobe Photoshop The background “empty” space of the image is selected through the Magic Wand tool. The number of pixels for this selection is shown in the red box. This value is then subtracted from the total pixel count of the image to determine the pixel count for the cell area.

Quantify the contraction phenotype by taking the mean ± SEM of the relative culture plate area covered by cells in four randomly selected frames per well. Analyze the images using Adobe Photoshop (Figure 6). Use the Magic Wand tool to select “empty” sections of the image that do not contain cells and subtract this pixel value from total pixels. Normalize this value by using the control DMSO treated well allowing for quantification of the difference in area taken up by cells before and after contraction. Perform at least 2 biological replicates and determine statistical significance by using a 2-way ANOVA test followed by Sidak’s multiple comparisons test.

### 3D contraction assay using BJ-5ta cells

Measure contraction in each well by using ImageJ. Specifically, measure the collagen matrix diameter by taking the average of two perpendicular diameter measurements of each matrix. Average these two measurements to calculate the average diameter of the collagen matrix. Then subtract the average diameter of the matrix after contraction (t = 30) from the average diameter before contraction (t = 0) to find the extent of contraction. Determine statistical significance by using a 2-way ANOVA test followed by Sidak’s multiple comparisons test.

## Limitations

This current protocol has been optimized for the analysis of sphingosine-1-phosphate mediated contraction in NIH3T3 cells in 2D contraction and in BJ-5ta cells in 3D contraction. We have also used this protocol to investigate lysophosphatidic acid (LPA) mediated contraction. The use of another pro-contractile signaling molecule or cell line would have to be further optimized. In addition, NIH3T3 fibroblast cells were not able to be used in the 3D contraction assay, as we experienced issues with polymerization with S1P.

## Troubleshooting

### Problem 1

**Figure 7:**
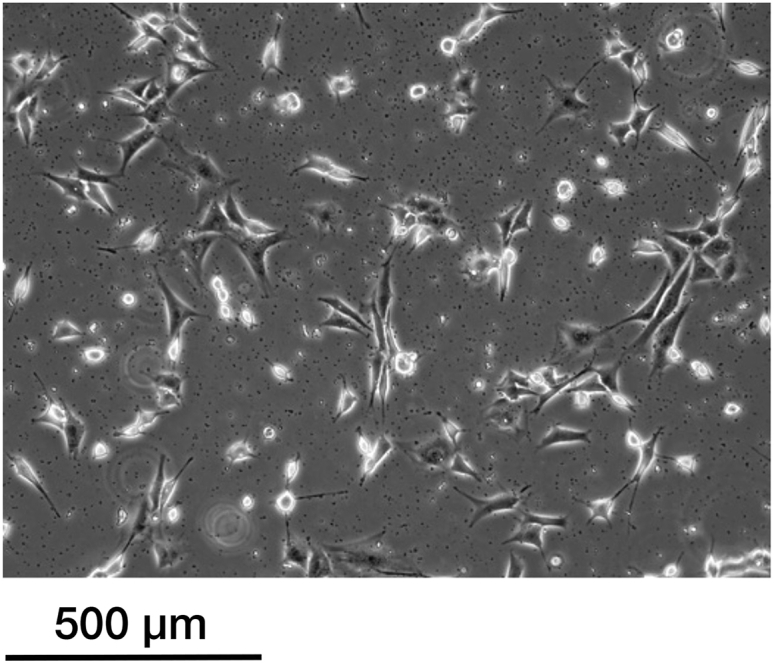
Troubleshooting unhealthy NIH3T3 cells An image of dead NIH3T3 cells and debris after treatment of the cells with an inhibitor.

Too many dead cells and cell debris in NIH3T3 cells after treatment with inhibitors (Figure 7; 2D Contraction assay using NIH3T3 cells, step 2).

### Potential solution

The NIH3T3 cell culture may not be in good condition. Cells may die if they are overgrown or if nutrients are depleted. Make sure not to overgrow cells and to wait at least 2 passages after thawing before treatment with inhibitors.

### Problem 2

**Figure 8:**
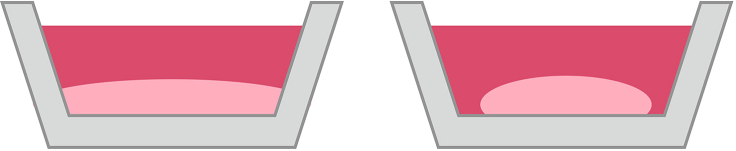
Troubleshooting the polymerization process The diagram on the left shows unsuccessful polymerization. The diagram on the right shows successful polymerization of the cell collagen solution after 2 days of incubation.

The cell collagen solution does not polymerize after 2 days of incubation (Figure 8; 3D contraction assay using BJ-5ta cells, step 10).

### Potential solution

The concentration of cells used in the collagen solution may be too low. Increase the amount of concentration of cells used. In addition, try incubating the cell collagen solution for an additional day, for a total of 3 days, to promote polymerization. Also, collagen is sensitive to changes in pH which can occur due cell death or contamination.

### Problem 3

Running out of cell collagen solution when plating out 500 μL into 30 wells (3D contraction assay using BJ-5ta cells, step 9).

### Potential solution

Because the solution is very viscous, some volume may be lost during pipetting. Next time, try plating out a smaller volume in each well. For example, plating 450 μL of cell collagen solution should still polymerize properly in the well for the experiment.

### Problem 4

Some wells polymerize during the 3D contraction assay and some wells do not (3D contraction assay using BJ-5ta cells, step 10).

### Potential solution

This can occur if the cell collagen solution is not mixed thoroughly enough. The concentration of cells will not be homogenous, allowing some wells to polymerize while other wells fail. When creating the cell collagen solution, use a serological pipette controller to pipette up and down and mix the solution well. Do not vortex, and mix carefully to avoid bubbles. Because of the viscosity of the solution, some volume may be lost.

## Resource availability

### Lead contact

Further information and requests for resources and reagents should be directed to and will be fulfilled by the lead contact, Matthew Pratt (matthew.pratt@usc.edu).

### Materials availability

This protocol did not generate new unique reagents.

## Data Availability

This protocol did not generate datasets or code.
